# Phylogenetic analyses and morphological characteristics support the description of a second species of *Tridimeris* (Annonaceae)

**DOI:** 10.3897/phytokeys.74.10371

**Published:** 2016-11-08

**Authors:** Andres Ernesto Ortiz-Rodriguez, Marcos Alberto Escobar-Castellanos, Miguel Angel Pérez-Farrera

**Affiliations:** 1 Departamento de Biologia Evolutiva, Instituto de Ecologia A.C., Xalapa, Veracruz, Mexico; 2 Instituto de Ciencias Biológicas, Universidad de Ciencias y Artes de Chiapas, Tuxtla Gutierrez, Chiapas, Mexico

**Keywords:** Dimery, Neotropical, Miliuseae, tropical rainforest

## Abstract

Based on phylogenetic and morphological evidence, *Tridimeris chiapensis* Escobar-Castellanos & Ortiz-Rodr., **sp**. **n.** (Annonaceae), a new species from the karst forest of southern Mexico, is described and illustrated. The new species differs from *Tridimeris hahniana*, the only described species in the genus, in that the latter has flowers with sepals densely tomentose outside, one (rarely two) carpel(s) per flower and fruits densely covered with golden-brown hairs, while *Tridimeris chiapensis* has flowers with glabrous sepals outside, two to five carpels per flower and glabrous fruits. Furthermore, a shallow triangular white patch at the base of the inner petals is found in *Tridimeris chiapensis*, a morphological character shared with the sister genus *Sapranthus* but absent in *Tridimeris hahniana*. Geographically, both species occur allopatrically. With just one known locality and seven individuals of *Tridimeris chiapensis* recorded in one sampling hectare, and based on application of the criteria established by the IUCN, we conclude tentatively that the species is critically endangered.

## Introduction

Annonaceae is a plant family composed of about 110 genera and 2,500 species of trees and lianas (Couvreur et al. 2012, Erkens et al. 2012). Most genera of Annonaceae (except *Asimina*, endemic to the USA) are exclusively tropical and many are important floristic elements in several lowland forests. Within continents, the endemism at generic level is very high and only the genus *Xylopia* is pantropical (Doyle and Le Thomas 1997). Recent phylogenetic analyses (Richardson et al. 2004, Chatrou et al. 2012) show that Annonaceae is composed of four major lineages, and on that basis, the family is now classified into four subfamilies: Anaxagoreoideae, Ambavioideae, Annonoideae and Malmeoideae. Of these, Annonoideae (50 genera and 1600 species) and Malmeoideae (50 genera and 700 species) are the most species-rich lineages in Annonaceae (Pirie and Doyle 2012). In Mexico the family is represented by eleven genera, *Anaxagorea*, *Annona*, *Cymbopetalum*, *Desmopsis*, *Guatteria*, *Mosannona*, *Sapranthus*, *Stenanona*, *Tridimeris*, *Unonopsis* and *Xylopia*, of which *Tridimeris* is endemic.

**Figure 1.**
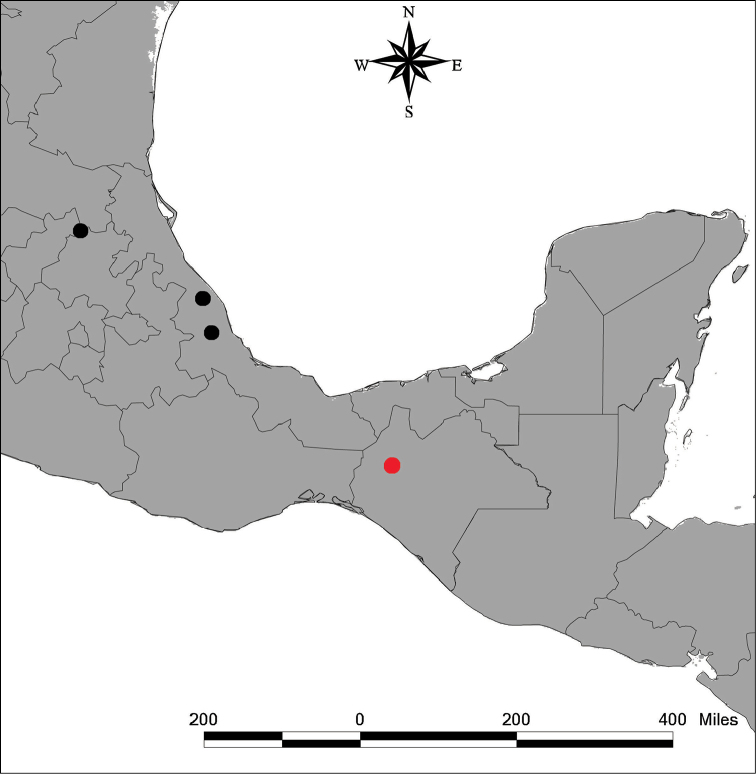
Distribution range of *Tridimeris chiapensis* Escobar-Castellanos & Ortiz-Rodr. (red dot) and *Tridimeris hahniana* Baill (black dots).

*Tridimeris* is a monotypic and poorly studied genus. Baillon (1869) described its only species, *Tridimeris hahniana* Baill., based on exemplars from Veracruz, Mexico (Turner 2013). The species, restricted to eastern Mexico in the states of San Luis Potosí, Puebla and Veracruz (Figure 1), is easily recognizable by its greenish and dimerous flowers (two sepals and four petals) and its large and fleshy fruits densely covered with golden-brown hairs (Schatz 1987). Dimery is uncharacteristic in Neotropical Annonaceae and it has been recorded only in *Anaxagorea silvatica* R. E. Fr., *Ephedranthus dimerus* J. C. Lopes, Chatrou & Mello-Silva and, *Malmea dimera* Chatrou (Lopes et al. 2014). Phylogenetic analyses based on molecular characters (Saunders et al. 2011, Chaowasku et al. 2012, 2014, Xue et al. 2011, 2014, Ortiz-Rodriguez et al. 2016) show that *Tridimeris hahniana* belongs to the Malmeoideae tribe Miliuseae, where along with *Desmopsis*, *Sapranthus* and *Stenanona* it forms the subtribe Sapranthinae (Ortiz-Rodriguez et al. 2016). *Sapranthus* and *Tridimeris* are closely related and together form the sister group of the remaining members of Sapranthinae. Although *Sapranthus* and *Tridimeris* have contrasting floral characteristics, with *Tridimeris* showing axillary, dimerous and greenish flowers whilst *Sapranthus* shows leaf-opposed, trimerous and usually brown to purple colored flowers, both genera are characterized by large and fleshy fruits, these last characteristics being their most obvious synapomorphy (Schatz 1987).

During a floristic study in southern Mexico, several individuals of an unusual species of Annonaceae were collected. The general characteristics of its flowers, notably dimery, suggested that it probably was a species related to the genus *Tridimeris*; however, its fruit characteristics did not fit with those of *Tridimeris hahniana*. To elucidate this, we performed molecular phylogenetic analyses including one sample of the putative new species and studied its morphological characteristics in detail to corroborate its identity at the genus level and to determine whether the collections from Chiapas represent a second species of *Tridimeris* for the Mexican flora.

## Material and methods

### Molecular data

DNA extraction was performed using a CTAB (acetyl trimethyl ammonium bromide) method (Doyle and Doyle 1987). Four plastid markers, *matK*, *rbcL* and *ycf1* coding regions and *trnL-F* spacer, were amplified and sequenced using the following primers: matK-13F/515R, matK-424F/788F and matK-449F/824R (Su et al. 2008); 1F/724R (Olmstead et al. 1992) and 636F/1460R (Fay et al. 1997; Fay et al. 1998); 72F/1674R and 914F/2323R (Chaowasku et al. 2012); trnL(UUA)/trnF(GGA) (Taberlet et al. 1991). For amplification each 14 µL PCR contained 2.02 µL of 5' buffer (Promega, Madison, WI, USA), 2.02 mL MgCl_2_ (25 mM), 1.02 µL dNTPs mix (8 mM), 0.22 µL of each primer (10 µM), 0.10 µL Taq polymerase (5U/µL) (Promega), 0.56 µL of BSA (Promega), 2 µL of template DNA, and finally dH_2_O added to bring to volume. The PCR program used comprised 35 cycles of 94°C for 45 s, 53 °C–65 °C for 30 s (annealing temperatures depending on each primer pair), 72°C for 2 min, with the initial denaturation for 3 min at 94°C and a final extension for 7 min. at 72°C. PCR products were purified with the QIAquick PCR Purification kit (Qiagen) and sequenced using the BigDye Terminator Cycle Sequencing kit (Applied Biosystems, Foster City, California, USA). The products were analyzed on a 310 automated DNA sequencer (Applied Biosystems) at the University of Washington High Throughput Genomics Unit, Seattle, Washington. The sequences obtained were first edited and assembled in Sequencher ver. 4.1 (Gene Codes Corp., Ann Arbor, MI, USA), and subsequently aligned using PhyDE-1 ver. 0.9971 (Müller et al. 2010). Additionally, sequences of the coding region *matK*, *ndhF*, *rbcL* and *ycf1* and *psbA-trnH* and *trnL-F* spacers of other Neotropical genera of Miliuseae (*Desmopsis*, *Sapranthus*, *Stenanona* and *Tridimeris hahniana*) and a few representatives of Asian Miliuseae were obtained from GenBank and included in the molecular matrix. [The samples, localities and GenBank accession numbers are listed in Appendix 1].

### Phylogenetic analyses

Phylogenetic relationships among taxa were estimated using Bayesian inference (BI), maximum likelihood (ML), and parsimony methods. Analyses of six cpDNA regions were conducted separately (for regions with information available for the new species) and in combination.

For BI, three partitioning strategies were used: (1) data matrix divided into six partitions based on DNA region identity, (2) six plastid markers concatenated and analyzed without partitioning, and (3) 2-partitioned, distinguishing coding (*matK*, *ndhF*, *rbcL* and *ycf1*) and non-coding (*psbA-trnH* and *trnL-F*) regions. jModelTest ver. 3.06 (Posada 2008) was used to determine the appropriate DNA substitution model and gamma rate heterogeneity for each partition using the Akaike Information Criterion (AIC). The BI analysis was performed using MrBayes ver. 3.2.1 (Ronquist and Huelsenbeck 2003) with two independent runs of four chains and ten million generations with trees sampled every 1000 generations. Convergence was assessed using the standard deviation of split frequencies as a convergence index, with values <0.01 interpreted as indicating good convergence. Tracer ver. 1.5 (Rambaut and Drummond 2007) was used to determine whether the parameter samples were drawn from stationary, unimodal distribution, and whether adequate effective sample sizes (ESS) for each parameter (ESS>200) were reached. The initial 25% of samples of each MCMC run were discarded as burn-in, and the remaining trees were summarized as posterior probabilities; PP values ≥ 0.95 were considered to represent strong support. Analysis performance of each partitioning strategy was assessed using Bayes factor. Bayes factors were calculated from the estimated harmonic means of likelihood using the sump command in MrBayes. Decisions were taken based on the 2ln Bayes factor criterion (Kass and Raftery, 1995), for which the Bayes factor scale of strength of evidence in favor of one hypothesis is: 0–2 (not worthy of mention), >2–6 (positive), >6–10 (strong), >10 (very strong).

For the ML analysis, the dataset was divided based on Bayes factor results (see above and results). Phylogenetic reconstruction was performed using RAxML ver. 8.2.4 (Stamatakis 2014) under the general time-reversible (GTR) nucleotide substitution model and 1000 non-parametric bootstraps using the CIPRES Science Gateway (Miller et al. 2010). Bootstrap support values were interpreted as weak (50–70%), moderate (71–80%) and strong support (81–100%).

The most parsimonious trees were obtained using the ratchet strategy (Nixon 1999) in Winclada ver. 1.0000 (Nixon, K. C. 1999–2002), running NONA ver. 2.0 (Goloboff 1993) on a combined dataset of six plastid regions, with nucleotide characters treated as unordered and equally weighted, 1000 iterations, holding 10 trees per iteration with 10% of nodes constrained, and all other parameters set to default. Branch support was assessed using bootstrap resampling, 1000 bootstrap-resampled pseudoreplicate matrices were each analyzed using 100 random addition sequences (multi*100). Ten trees were retained during TBR swapping after each search initiation (hold/10) using NONA ver. 2.0 and performed in WinClada, with the same interpretations of support level as in the ML analyses.

### Morphological differentiation

We examined the specimens of *Tridimeris hahniana* deposited at XAL herbarium (Thiers 2016). Also, we consulted the digitized type specimens available at JSTOR Global Plants (http://plants.jstor.org/). The putative new species was recognized using the unique combination of features criteria (Donoghue 1985) through comparisons with morphologically similar species and literature review (Schatz 1987). Finally, we elaborated the species description following terminology presented in Hickey (1973).

### Conservation status

We assessed the conservation status by calculating the extent of occurrence (EOO) and the area of occupancy (AOO) using the GeoCAT tool (Bachman et al. 2011) and applying the IUCN Red List Categories and criteria (IUCN 2001).

## Results

### Analysis of individual cpDNA regions

Each individual cpDNA region provided a relatively good resolution within Sapranthinae clade, with most branches resolved in the four separate trees (Suppl. material 1–4). Analyses of the *matK*, *rbcL* and *ycf1* coding regions showed that Sapranthinae is composed of two main subclades, the *Desmopsis*-*Stenanona* clade and the *Sapranthus*-*Tridimeris* clade, while the analysis of *trnL-F* spacer showed very low resolution recovering only the *Sapranthus*-*Tridimeris* clade (Suppl. material 1–4). Each phylogenetic hypothesis unequivocally placed the new species within the *Sapranthus*-*Tridimeris* clade.

### Analysis of combined data

The concatenated 32-accession dataset contained 6419 aligned positions, of which 746 were variable and 208 were parsimony informative. For the Bayesian analyses, the substitution model was GTR+G for *matK*, *trnL-F*, *psbA-trnH* and unpartitioned datasets, GTR+G+I for *rbcL*, *ndhF* and coding datasets and HKY+G for non-coding dataset.

The six partitioned strategy considerably improved the mean −lnL values in the Bayesian analyses (mean −lnL non-partitioned = –15754.57; mean –lnL 2-partitioned= –15725.73; mean –lnL 6-partitioned = –15722.11). Bayes factor comparison indicated that the analyses using six partitions provided a better explanation of the data than unpartitioned and 2-partitioned analyses. For the ML analyses the likelihood score of the optimal ML tree, was *ln L* = –15572.87. The parsimony analysis of the combined regions resulted in 20 most parsimonious trees of 1030 steps with a Consistence Index of 0.79 and a Retention Index of 0.60. The subsequent presentation of the results is restricted to the 50% majority rule consensus tree derived from Bayesian analyses using six partitions.

**Figure 2.**
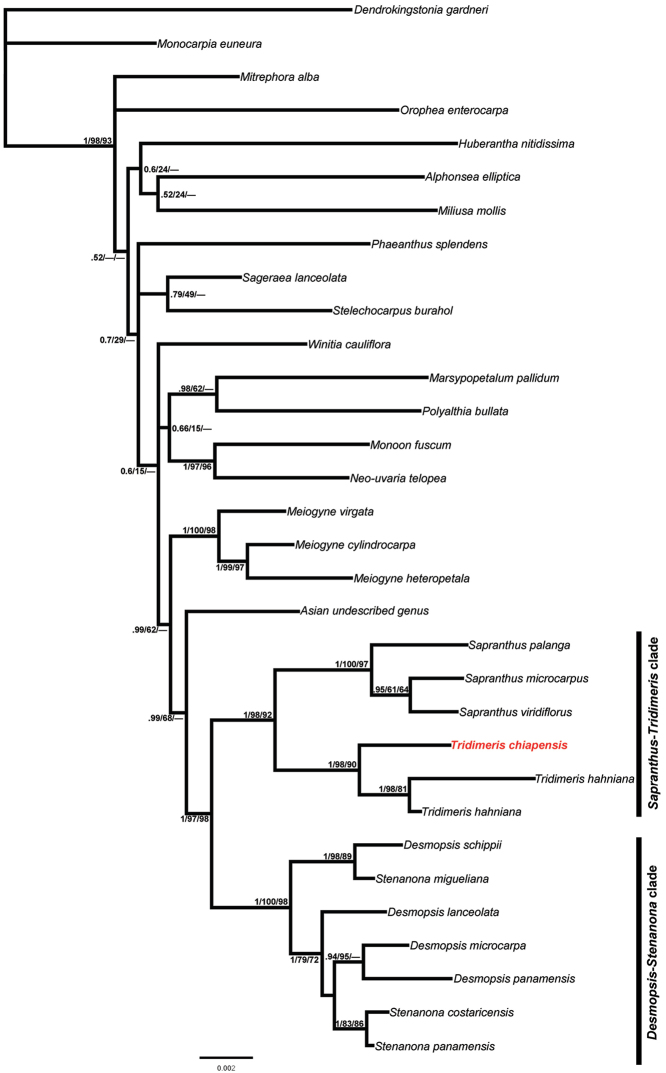
The 50% majority-rule consensus tree from the Bayesian analysis of six cpDNA markers. Numbers on branches of the major clades indicate Bayesian posterior probabilities (PP), maximum likelihood (MLBS) and parsimony (MPBS) bootstrap values in that order. In red, the position of *Tridimeris chiapensis* Escobar-Castellanos & Ortiz-Rodr.

The partitioned BI, ML and parsimony analyses resulted in similar tree topologies. The 50% majority-rule consensus BI tree resulting is shown in Fig. 2. All phylogenetic analyses indicate that the Neotropical Miliuseae (Sapranthinae subtribe) forms two strongly supported clades: the *Desmopsis*-*Stenanona* clade (1.0 PP, 100% bootstrap support (MLBS), 98% MP bootstrap support (MPBS)) and the *Sapranthus*-*Tridimeris* clade (1.0 PP, 98% MLBS, 92% MPBS). The first clade includes the genera *Desmopsis* and *Stenanona*, however, they appear to be not monophyletic and species of both genera appear intermingled. The second clade is composed of *Sapranthus* and *Tridimeris*, with each resolved as monophyletic group (*Sapranthus*, 1.0 PP, 98% MLBS, 97% MPBS; *Tridimeris*, 1.0 PP, 100% MLBS, 90% MPBS). The phylogenetic hypothesis indicates that effectively the new species, *Tridimeris chiapensis*, is part of the genus *Tridimeris*.

### Morphological differentiation

**Figure 3.**
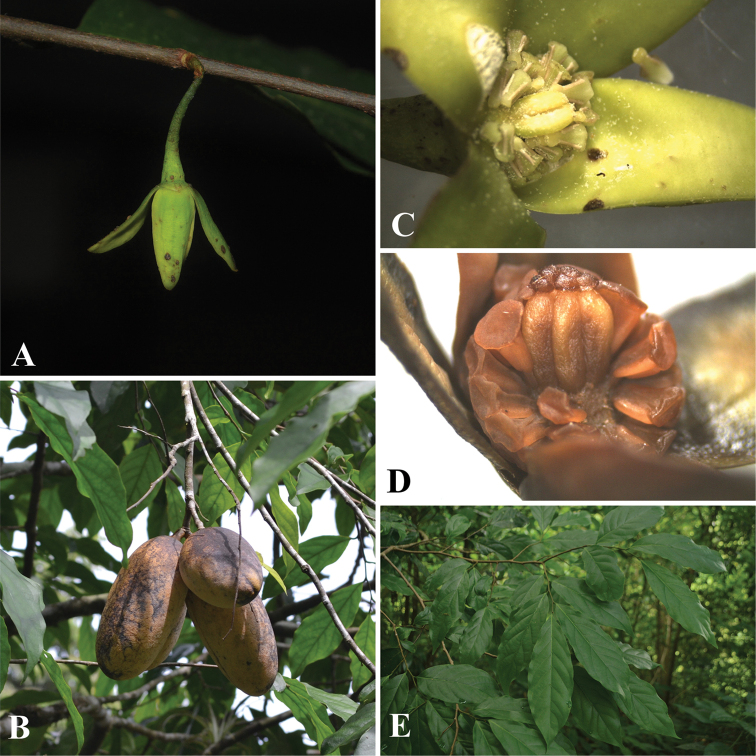
*Tridimeris chiapensis* Escobar-Castellanos & Ortiz-Rodr. **A** Dimerous flower **B** Large and fleshy fruits **C** Flower showing the pollen release and a triangular white patch at the base of the inner petals **D** Five carpels surrounded by numerous stamens **E** Leafy branches. Photographs by Marcos Escobar-Castellanos.

Morphologically, *Tridimeris chiapensis* has a set of morphological characters that clearly distinguish it from *Tridimeris hahniana* (Fig. 3, Table 1). In addition, both species occur in disjunct locations and therefore distributed allopatrically (Fig. 1).

**Table 1.** Comparison of diagnostic morphological characters of *Tridimeris chiapensis* and *Tridimeris hahniana*.

Characters	*Tridimeris chiapensis*	*Tridimeris hahniana*
Pedicel	Glabrous	Golden tomentose
Sepals	Glabrous outside	Densely tomentose outside
Inner petals	Thick and fleshy	Flat and thin
Carpels	2–5	1 (occasionally 2)
Monocarps	Glabrous	Golden brown tomentellous
Distribution	Mexico (Chiapas)	Mexico (Puebla, San Luís Potisí and Veracruz)

## Discussion

The phylogenetic analyses showed that *Tridimeris chiapensis* and *Tridimeris hahniana* form a strongly supported monophyletic group (Fig. 2). The two species of *Tridimeris* share axillary inflorescences, dimery flowers (two sepals and four petals), greenish petals, and large and fleshy fruits. Furthermore, both species have pocket domatia in the axils of secondary veins. As in previous studies, *Tridimeris* and *Sapranthus* appear to be closely related (Saunders et al. 2011, Chaowasku et al. 2012, 2014, Xue et al. 2011, 2014, Ortiz-Rodriguez et al. 2016) and together form the *Sapranthus*-*Tridimeris* clade.

*Tridimeris chiapensis* clearly differs from *Tridimeris hahniana* by its number of carpels per flowers, fruit surface, glabrous pedicels and sepals, and by the presence of a triangular white patch near the base of inner petals (Fig. 3). A similar white patch is found in the inner petals of *Sapranthus viridiflorus* G.E. Schatz, which have been considered by Schatz (1998) as a vestigial food body since food bodies are morphological modifications of a specific area of inner petals as food reward for floral visitors (Schatz 1987). However, a more detailed study of these structures is needed, as well as the compounds present in this structure and its anatomical characteristics in order to determine its function.

Ecologically *Tridimeris chiapensis* inhabits wet forests on karstic topography around 1000 m elevation, while *Tridimeris hahniana* occurs in lowland wet forests (200–900 m) or even in cloud forests in the northern portion of its distribution (Schatz 1987). The type locality of *Tridimeris chiapensis*, the protected natural area La Pera in Chiapas, is a karstic zone covered mostly by tropical rain forest. This area among other similar regions of southern Mexico, are considered centers of plant endemism (Wendt 1987).

## Taxonomic treatment

### Tridimeris chiapensis

Taxon classificationPlantaeMagnolialesAnnonaceae

Escobar-Castellanos & Ortiz-Rodr. sp. nov.

urn:lsid:ipni.org:names:77158527-1

Figs 1 , 2 , 3

#### Type.

Mexico. Chiapas, Municipio de Berriozábal, Zona Sujeta a Protección Ecológica “La Pera”, Campamento “Trepatroncos” carretera Berriozábal-Joaquín Miguel Gutiérrez, km. 12 desvío a Montebello, 1081 m, 16°52'20.3"N, 93°19'32.5"W, 11 August 2016 (fl) *Escobar-Castellanos M. A. 0689* (holotype HEM; isotypes: XAL, MO).

#### Diagnosis.

*Tridimeris chiapensis* is phylogenetically related to *Tridimeris hahniana* with which it shares axillary and dimerous flowers and large and fleshy fruits. However, *Tridimeris chiapensis* differs in having flowers with glabrous sepals, a triangular white patch near the base of inner petals and 2-5 carpels per flower and glabrous fruits (Fig. 3), while *Tridimeris hahniana* has flowers with sepals densely tomentose outside, 1 or 2 carpels per flower and fruits densely covered with golden-brown hairs.

Tree 3–9 m tall and 3–14 cm DBH; young branches slightly pubescent, trichomes appressed and golden-brown in color, glabrescent with age. **Leaves** membranaceous to chartaceous, alternate, phyllotaxy distichous, 11–20 cm long to 3.5–8 wide, narrowly elliptic to obovate, the apex acute to acuminate, the base acute to obtuse, sometimes asymmetrical; upper surface glabrous, the lower side glabrescent; venation brochidodromus, 6–9 veins per side, pocket domatia in the axils of the main veins; the midrid impressed above and slightly canaliculate toward the base (sometimes with erect to appressed light-brown hairs), lateral veins barely elevated above; the midrib and lateral veins prominently elevated below and with sparsely light-brown hairs, lateral veins decurrent at midrid insertion ; petiole swollen, 0.5–1 cm long, canaliculate, with sparsely light-brown hairs. **Inﬂorescences** always one-ﬂowered, axillary, sometimes arising on leafless part of branches (ramiflory), the pedicel glabrous, 1–1.7 cm long, bearing 2–3 minute, densely golden tomentose and broadly ovate basal bracts. **Sepals** 2, connate, to 2 mm long × 4–5 mm wide, decurrent along the pedicel, broadly ovate, rounded at apex, glabrous inside and outside, the margins ciliate. **Petals** 4, in two subequal whorls, 8–14 mm long × 3–5 mm wide, lanceolate to triangular, green to yellowish green, glabrous inside and outside, the margins ciliate, acute at apex, the base truncate and cusped around the stamens; the outer petals, more or less thin, with faint venation, reflexed at anthesis; the inner petals thicker and fleshier and not reflexed with a shallow, more or less triangular white patch near the base. **Stamens**, c.a. 40, 1–1.5 mm long, extrorse, filament very short, apical part of connective expanded over the thecae, shield-shaped, ellipsoid to angulate, glabrous. **Carpels**, 2–5 per flower, to 2.5 mm long; the stigma more or less globose and essentially glabrous; style absent; the ovaries ellipsoid and more or less curved, like a small banana with sparsely light-brown hairs; the ovules, 12–18, lateral, in two rows. **Monocarps**, 1-4 per fruit, large and fleshy, 8–11 cm long × 3–5 cm wide, ellipsoid, the apex and base rounded, glabrous, shortly stipitate, stipes to 7 mm long; young monocarps green, yellow to light brown when ripe with a peach-like sweet odor; seeds lunate to wedge-shaped, 1.3–2.2 cm long with lamellate ruminations.

#### Habitat and ecology.

The type locality of *Tridimeris chiapensis* is locally named as “La Pera” and “Pozo Turipache” or “El Pozo” and it lies within the ecological state reserve La Pera, which is mostly covered by tropical rainforest. Thin soils, rough limestone outcrops, caves, crevices, sinkholes and almost no surface water that form a typical karst landscape can be observed around El Pozo (Wake and Johnson 1989). Also, fogs forming cloudbanks are common throughout the year, though absent during the dry season (Wake and Johnson 1989).

*Tridimeris chiapensis* forms part of the understory vegetation and it is associated with *Mortoniodendron ocotense* Ishiki & T. Wendt, *Mortoniodendron vestitum* Lundell, *Trichilia moschata* Sw., *Neea tenuis* Standl., *Pseudolmedia glabrata* (Liebm.) C.C. Berg, *Quararibea funebris* (La Llave) Vischer, *Quercus lancifolia* Schltdl. & Cham. and *Heliocarpus appendiculatus* Turcz. (Escobar-Castellanos 2016).

#### Phenology.

The species was found in full bloom in August and bearing fruits in March and May.

#### Etymology.

The specific epithet is in honor of the Mexican state of Chiapas where the species was found.

#### Conservation status.

*Tridimeris chiapensis* is known only from the type locality at the ecological state reserve La Pera. According to the criteria established by the IUCN, it is possible to tentatively determine that the species is Critically Endangered [CR B1ab (iii)]. The Area of occupancy (AOO) of *Tridimeris chiapensis* is 0.314 km² and the Extent of occurrence (EOO) is 1.519 km², suggesting a very restricted overall distribution. Although the only known population of the species is located within a protected natural area, only 7 individuals of *Tridimeris chiapensis* in one hectare of sampling were recorded (Escobar-Castellanos 2016). The 3000 ha of La Pera’s rainforest estimated by Espinosa (2014) and its species are threatened by non-sustainable activities (logging, fires, illegal settlements) and forests in this region are fragmented and only some remnants persist which are surrounded by roads, croplands and cattle pastures (Medina et al. 2006, Luna-Reyes et al. 2015).

#### Additional specimens examined.

MEXICO. Chiapas, Municipio de Berriozábal: Zona Sujeta a Protección Ecológica “La Pera”, Campamento “Trepatroncos” carretera Berriozábal-Joaquín Miguel Gutiérrez, km. 12 desvío a Montebello, 1081 m, 16°52'20.3"N, 93°19'32.5"W, May 2014 (fr) *Escobar-Castellanos M. A. 0599* (HEM), May 2015 (fr) *Ortiz-Rodriguez A. E. 801* (XAL); Zona Sujeta a Protección Ecológica “La Pera”, Predio “La Selva”, desvío hacia San Joaquín, carretera Berriozábal-Joaquín Miguel Gutiérrez, Sistema kárstico, 14 km al NO de Berriozábal. Plot X, Tree No. 27, 1049 m, 19°52'50.45"N, 93°19'07.92"W, 11 August 2016 (fl) *Escobar-Castellanos M. A. 0690* (HEM); Zona Sujeta a Protección Ecológica “La Pera”, Predio “La Selva”, desvío hacia San Joaquín, carretera Berriozábal-Joaquín Miguel Gutiérrez, Sistema kárstico, 14 km al NO de Berriozábal, 1050 m, 19°52'54.62"N, 93°19'09.77"W, 31 March 2014 (fr) *Escobar-Castellanos M. A. 0556* (HEM).

## Supplementary Material

XML Treatment for Tridimeris chiapensis

## Appendix 1

Voucher specimens for the accessions cited in this study. The information is presented in the following order: Taxon; GenBank accessions: *rbcL*; *matK*; *ndhF*; *trnLF*; *psbA-trnH*; *ycf1*; Voucher information (herbaria in parentheses). Long dash (—) = sequence not available.

***Alphonsea elliptica*** Hook.f. & Thomson; AY318966; AY518807; JQ690401; AY319078; JQ690402; KJ418378; Van Balgooy 5141 (L); Gardner & Sidisunthorn ST 2214, Indonesia. **Asian undescribed GenusA**; —; KC857607; KC857608; KC857606; —; JX544757; Chaowasku 108(L) Thailand. ***Dendrokingstonia gardneri*** Chaowasku; KJ418381; KJ418391; KJ418385; KJ418406; KJ418399; KJ418378; Gardner & Sidisunthorn ST 2214 (L) Thailand. ***Desmopsis lanceolata*** Lundell; KU727378; KU727296; —; KU727414; KU727337; KY026102; Rubén Martínez Camilo 2370, (XAL) Mexico. ***Desmopsis microcarpa*** R.E. Fr.; AY319059; AY518804; JX544771; AY319173; AY841461; JX544758; Chatrou et al. 85 (U) Costa Rica. ***Desmopsis panamensis*** (B.L. Rob.) Saff.; GQ981723; GQ981981; —; —; GQ982207; —; R. Perez STRI: BCI 158574, no collection data, Panama. ***Desmopsis schippii*** Standl.; AY319060; AY518805; JQ723786; AY319174; —; —; Chatrou et al. 94 (U) Costa Rica, Alfaro 4572 (U) Costa Rica. ***Huberantha nitidissima*** (Dunal) Chaowasku; KF682103; JQ889989; KF682116; KF682105; KF709056; JQ889976; Ford & Metcalfe 4708 (HKU) Australia, Ford AF 4967, no collection data, Australia. ***Marsypopetalum pallidum*** (Blume) Kurz; AY318980; AY518834; —; AY319092; —; —; Kessler PK 3192 (L) Thailand. ***Meiogyne cylindrocarpa*** (Burck) Heusden; KF301029; AY518796; KF611914; KF573500; KJ418402; JQ723931; L. Raulerson & M. Mesngon 18331 (L) Guam, Ridsdale DV-M1-1930 (L) no locality data, Marler s.n. Tinian Island. ***Meiogyne heteropetala*** (F. Muell.) D.C.Thomas, Chaowasku & R.M.K.Saunders; JQ723853; AY773280; JQ723790; JQ723906; KC857559; JQ723927; Sankowsky 4140(BRI) Australia, Kemp TH 7267 (L) Australia. ***Meiogyne virgata*** (Blume) Miq.; AY318982; AY518798; JQ723805; AY319094; JX544784; JQ723945; Kessler PK 2751 (L) Indonesia. ***Miliusa mollis*** Pierre; AY318990; AY518851; JQ690503; AY319101; JQ690504; JQ690505; Kessler PK 3207 (L) Thailand. ***Mitrephora alba*** Ridl.; AY318994; AY518855; JQ723807; AY319106; JQ889978; JQ723947; Chalermglin 530706 (HKU) Thailand. ***Monocarpia euneura*** Miq.; AY318998; AY518865; AY841412; AY319111; AY841477; —; Slik 2931(L) Borneo. ***Monoon fuscum*** (King) B.Xue & R.M.K.Saunders; AY318973; AY518787; JX544779; AY319085; KM924989; JX544767; Kessler PK 3222 (L) Thailand. ***Neo-uvaria telopea*** Chaowasku; JX544755; JX544751; JX544778; JX544783; JX544791; JX544766; Chaowasku 77 (L) Thailand. ***Orophea enterocarpa*** Maingay ex Hook.f. & Thomson; AY319006; AY518815; JQ690416; AY319119; JQ690417; JQ690418; Chalermglin 440403 (TISTR) Thailand. ***Phaeanthus splendens*** Miq.; JX227921; AY518864; JX544777; AY319126; JX544790; JX544765; Kessler B 1564 (L) Indonesia. ***Polyalthia bullata*** King; JX227908; JX544825; JX544839; JX227859; JX544809; JX544818; P. Chalermglin 521115 (HKU) Thailand, Chaowasku 34 (L) Thailand. ***Sageraea lanceolata*** Miq.; AY319050; AY518799; JX544774; AY319164; JX544787; JX544762; Ridsdale DV-M2-1692 (L) Malaysia. ***Sapranthus microcarpus*** (Donn. Sm.) R.E. Fr.; AY319052; AY518806; —; AY319166; —; —; Maas et al. 8457 (U) Honduras. ***Sapranthus palanga*** R.E. Fr.; JQ590193; JQ586518; —; —; HG963548; —; Adrian Guadamuz BioBot00012, no collection data, Costa Rica. ***Sapranthus viridiflorus*** G.E. Schatz; AY319051; AY743493; AY841422; AY319165; AY841515; JQ723955; Chatrou et al. 55 (U) Costa Rica. ***Stelechocarpus burahol*** (Blume) Hook. f. & Thomson; AY319053; AY518803; JQ723814; AY319167; JX544788; JQ723956; Mols 13 (L) Indonesia, Karim s.n. (HKU), Singapore. ***Stenanona costaricensis*** R.E. Fr.; AY319069; AY518801; JX544772; AY319183; AY841516; JX544759; Chatrou et al. 67 (U) Costa Rica. ***Stenanona migueliana*** Ortiz-Rodr. & G.E. Schatz; KU727397; KU727316; —; KU727435; KU727358; KY026103; Andres E. Ortiz-Rodriguez 796 (XAL) Mexico. ***Stenanona panamensis*** Standl.; AY319070; AY518802; —; AY319184; —; —; Chatrou et al. 100 (U) Costa Rica. ***Tridimeris hahniana*** Baill.; AY319055; —; —; AY319169; —; —; Schatz 1198 (K), Mexico; JX544753; JX544750; JX544773; JX544782; JX544786; JX544761—; Maas 8646 (U) Mexico. ***Tridimeris chiapensis*** Escobar-Castellanos & Ortiz-Rodr.; KY026100; KU727329; —; KY026101; —; KY026104; Andres E. Ortiz-Rodriguez 801 (XAL) Mexico. ***Winitia cauliflora*** (Scheff.) Chaowasku; AY319054; AY518800; JX544776; AY319168; JX544789; JX544764; no collection data, Hort. Bot. Bog. XV-A-196 (L).

## Supplementary materials

Supplementary material 1 Figure S1. The 50% majority-rule consensus tree from the Bayesian analysis of *rbcL* coding region Data type: JPEG image Explanation note: Numbers on branches of the major clades indicate Bayesian posterior probabilities (PP), maximum likelihood (MLBS) and parsimony (MPBS) bootstrap values in that order. This dataset is made available under the Open Database License (http://opendatacommons.org/licenses/odbl/1.0/). The Open Database License (ODbL) is a license agreement intended to allow users to freely share, modify, and use this Dataset while maintaining this same freedom for others, provided that the original source and author(s) are credited.Andres Ernesto Ortiz-Rodriguez, Marcos Alberto Escobar-Castellanos, Miguel Angel Pérez-Farrera

Supplementary material 2 Figure S2. The 50% majority-rule consensus tree from the Bayesian analysis of *matK* coding region Data type: JPEG image Explanation note: Numbers on branches of the major clades indicate Bayesian posterior probabilities (PP), maximum likelihood (MLBS) and parsimony (MPBS) bootstrap values in that order. This dataset is made available under the Open Database License (http://opendatacommons.org/licenses/odbl/1.0/). The Open Database License (ODbL) is a license agreement intended to allow users to freely share, modify, and use this Dataset while maintaining this same freedom for others, provided that the original source and author(s) are credited.Andres Ernesto Ortiz-Rodriguez, Marcos Alberto Escobar-Castellanos, Miguel Angel Pérez-Farrera

Supplementary material 3 Figure S3. The 50% majority-rule consensus tree from the Bayesian analysis of *ycf1* coding region Data type: JPEG image Explanation note: Numbers on branches of the major clades indicate Bayesian posterior probabilities (PP), maximum likelihood (MLBS) and parsimony (MPBS) bootstrap values in that order. This dataset is made available under the Open Database License (http://opendatacommons.org/licenses/odbl/1.0/). The Open Database License (ODbL) is a license agreement intended to allow users to freely share, modify, and use this Dataset while maintaining this same freedom for others, provided that the original source and author(s) are credited.Andres Ernesto Ortiz-Rodriguez, Marcos Alberto Escobar-Castellanos, Miguel Angel Pérez-Farrera

Supplementary material 4 Figure S4. The 50% majority-rule consensus tree from the Bayesian analysis of *trnLF* spacer Data type: JPEG image Explanation note: Numbers on branches of the major clades indicate Bayesian posterior probabilities (PP), maximum likelihood (MLBS) and parsimony (MPBS) bootstrap values in that order. This dataset is made available under the Open Database License (http://opendatacommons.org/licenses/odbl/1.0/). The Open Database License (ODbL) is a license agreement intended to allow users to freely share, modify, and use this Dataset while maintaining this same freedom for others, provided that the original source and author(s) are credited.Andres Ernesto Ortiz-Rodriguez, Marcos Alberto Escobar-Castellanos, Miguel Angel Pérez-Farrera
