# Supplementary figures and images for: Phylogenetic analyses and morphological characteristics support the description of a second species of Tridimeris (Annonaceae)
Source: PhytoKeys. 2016 Nov 8;(74):79–95. doi: 10.3897/phytokeys.74.10371 (PMC5234549; doi:10.3897/phytokeys.74.10371)

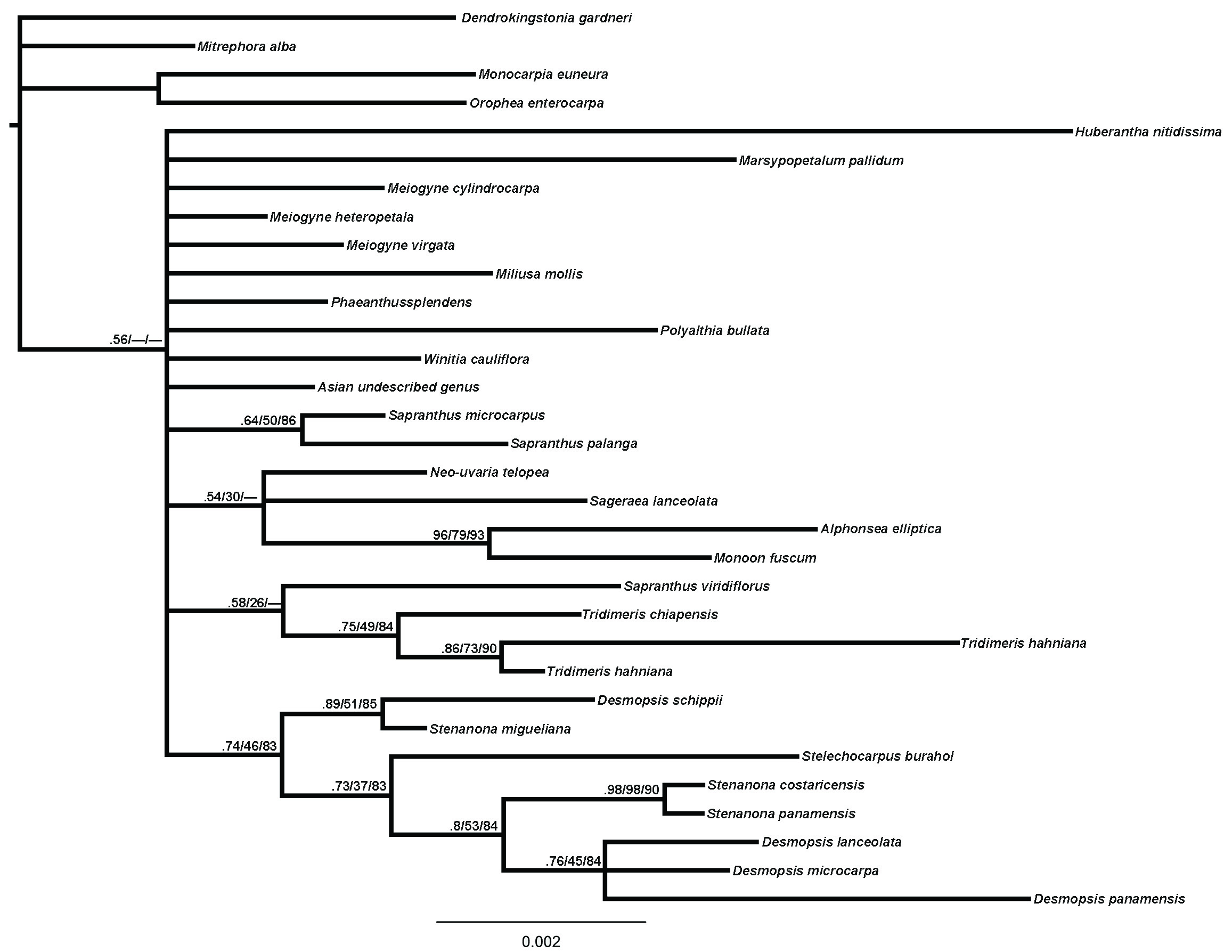

Supplement: Supplementary material 1 — Figure S1. The 50% majority-rule consensus tree from the Bayesian analysis of rbcL coding region [file phytokeys-074-079-s001.jpg]

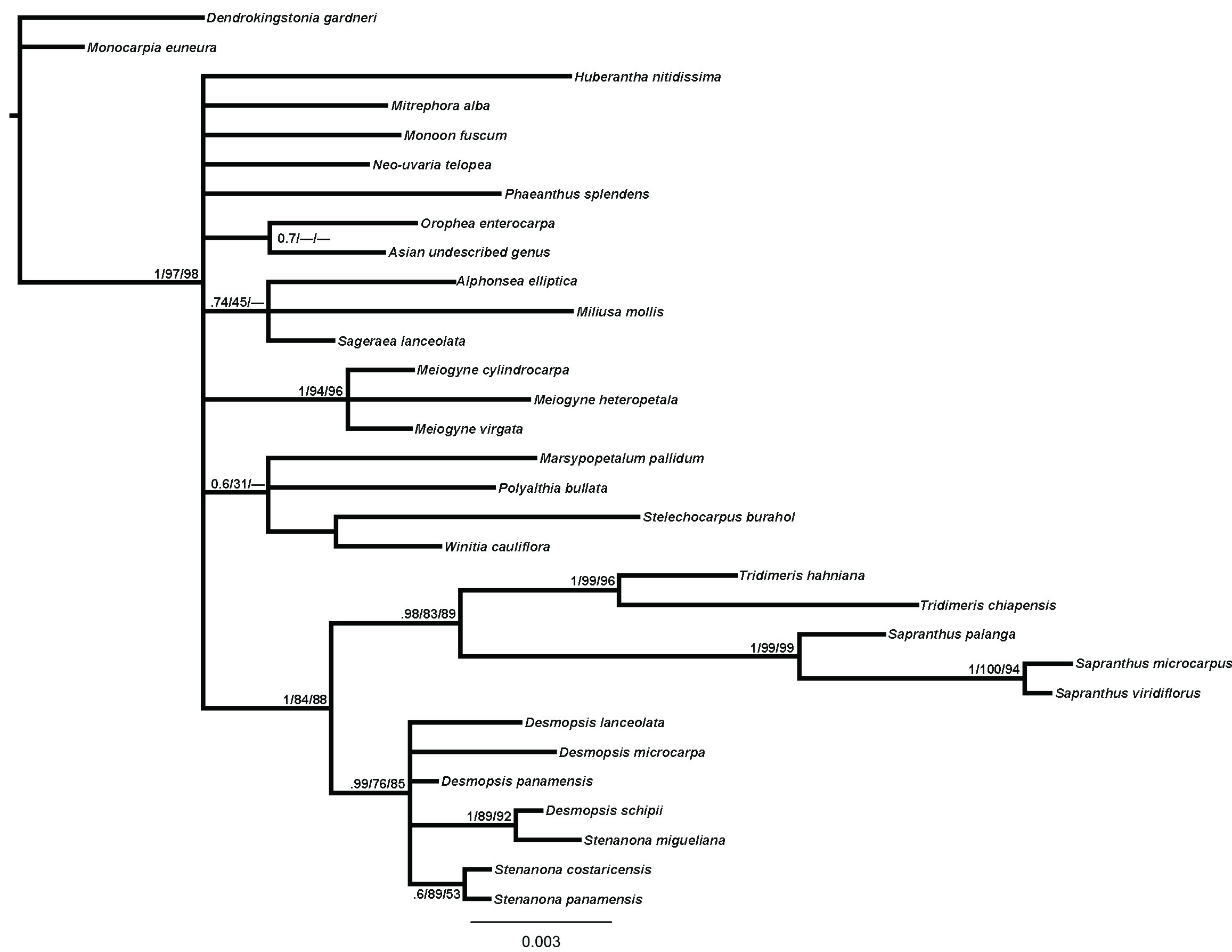

Supplement: Supplementary material 2 — Figure S2. The 50% majority-rule consensus tree from the Bayesian analysis of matK coding region [file phytokeys-074-079-s002.jpg]

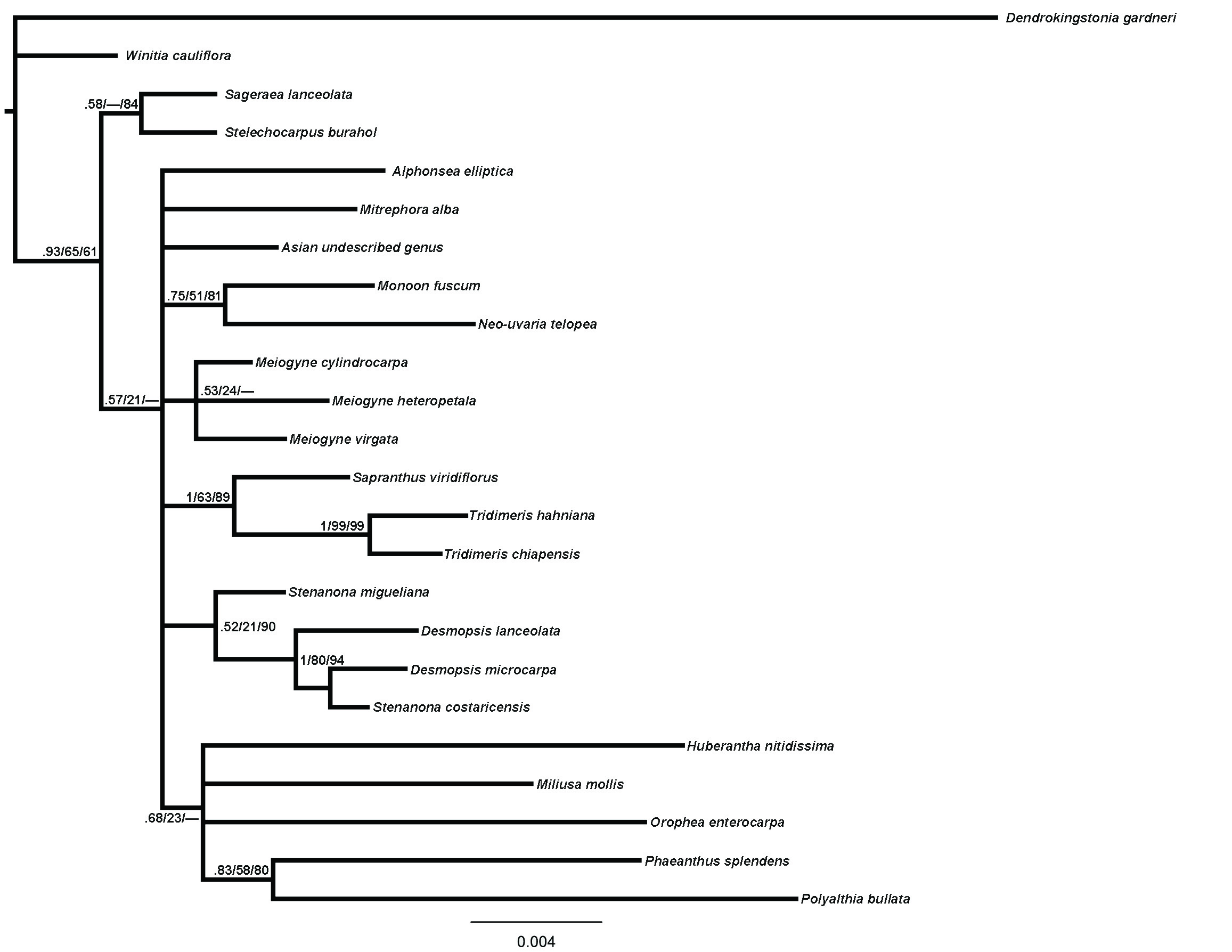

Supplement: Supplementary material 3 — Figure S3. The 50% majority-rule consensus tree from the Bayesian analysis of ycf1 coding region [file phytokeys-074-079-s003.jpg]

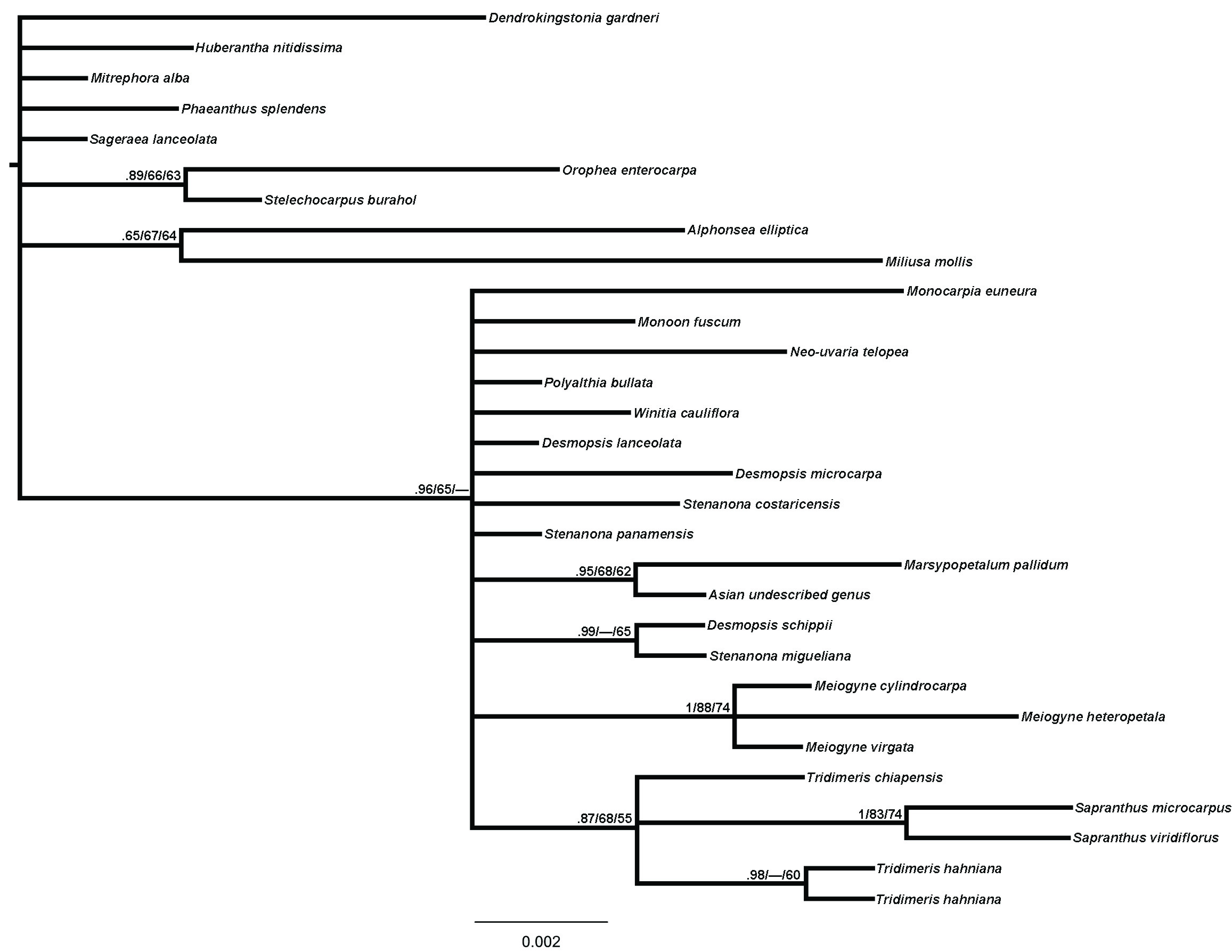

Supplement: Supplementary material 4 — Figure S4. The 50% majority-rule consensus tree from the Bayesian analysis of trnLF spacer [file phytokeys-074-079-s004.jpg]
